# Modern Processing of Indian Millets: A Perspective on Changes in Nutritional Properties

**DOI:** 10.3390/foods11040499

**Published:** 2022-02-09

**Authors:** N. A. Nanje Gowda, Kaliramesh Siliveru, P. V. Vara Prasad, Yogita Bhatt, B. P. Netravati, Chennappa Gurikar

**Affiliations:** 1 Department of Food Technology, Faculty of Life and Allied Health Sciences, Ramaiah University of Applied Sciences, Bangalore 560054, India; yogitabhatt.ft.ls@msruas.ac.in (Y.B.); netrapalled@gmail.com (B.P.N.); chennappa.ft.ls@msruas.ac.in (C.G.); 2 Department of Grain Science and Industry, Kansas State University, Manhattan, KS 66506, USA; 3 Department of Agronomy, Kansas State University, Manhattan, KS 66506, USA; vara@ksu.edu

**Keywords:** millets, processing, nutrients, dietary fiber, pearl, foxtail

## Abstract

Globally, billions of people are experiencing food insecurity and malnutrition. The United Nations has set a global target to end hunger by 2030, but we are far from reaching it. Over the decade, climate change, population growth and economic slowdown have impacted food security. Many countries are facing the challenge of both undernutrition and over nutrition. Thus, there is a need to transform the food system to achieve food and nutrition security. One of the ways to reach closer to our goal is to provide an affordable healthy and nutritious diet to all. Millets, the nutri-cereals, have the potential to play a crucial role in the fight against food insecurity and malnutrition. Nutri-cereals are an abundant source of essential macro- and micronutrients, carbohydrates, protein, dietary fiber, lipids, and phytochemicals. The nutrient content and digestibility of millets are significantly influenced by the processing techniques. This review article highlights the nutritional characteristics and processing of Indian millets, viz. foxtail, kodo, proso, little, and pearl millets. It also envisages the effect of traditional and modern processing techniques on millet’s nutritional properties. An extensive literature review was conducted using the research and review articles related to processing techniques of millets such as fermentation, germination, dehulling, extrusion, cooking, puffing, popping, malting, milling, etc. Germination and fermentation showed a positive improvement in the overall nutritional characteristics of millets, whereas excessive dehulling, polishing, and milling resulted in reduction of the dietary fiber and micronutrients. Understanding the changes happening in the nutrient value of millets due to processing can help the food industry, researchers, and consumers select a suitable processing technique to optimize the nutrient value, increase the bioavailability of nutrients, and help combat food and nutrition security.

## 1. Introduction

Millets are termed as “yesterday’s coarse grains and today’s nutri-cereals.” Millets are considered to be “future crops” as they are resistant to most of the pests and diseases and adapt well to the harsh environment of the arid and semi-arid regions of Asia and Africa [1]. Millets are small-seeded grains, the most common and important for food being sorghum (*Sorghum bicolor* L.), pearl millet (*Pennisetum glaucum*), finger millet (*Eleusine carocana*), teff (*Eragrostis tef*), proso millet (*Panicum miliaceum*), kodo millet (*Paspalum scrobiculatum*), foxtail millet (*Setaria italica*), little millet (*Panicum sumatrense*) and fonio (*Digitaris exilis*) [1]. After decades of negligence, nutri-cereals are making a strong comeback in the Indian cereal’s production segment. India dominates the global production of millets with a total share of about 40.62% and an estimated production of about 10.91 million tonnes during 2018–2019 [2]. Although India ranks first in nutri-rich millet production and second in rice and pulses across the globe, it also—unfortunately—ranks second in child malnutrition incidences. India is home to more than one-third of the world’s malnourished children [3]. By contrast, the country has also become a hub for diabetic and overweight populace, putting the country under a double burden of malnutrition [4]. The majority of millets are three to five times more nutritious than most cereals (rice, *Oryza sativa*; wheat, *Triticum aestivum*; maize, *Zea mays*) in terms of vitamins, fiber, proteins, and minerals (calcium and iron) and are gluten-free; hence, they are known as “superfoods” [2]. The nutri-rich millets are the viable solution to reduce the rising incidences of malnutrition and metabolic disorders and can enhance the nutrition and food security of the country.

**Figure 1:**
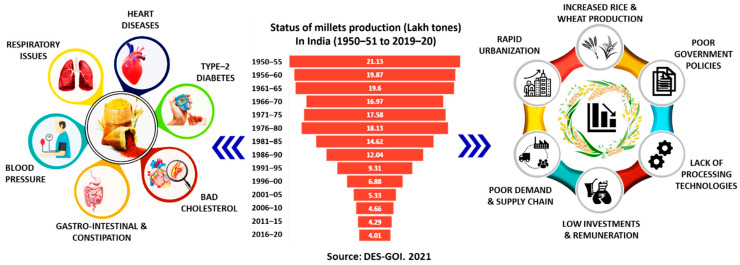
Millets: health benefits, production, and challenges in India. Data taken from various issues [17].

Millets are a highly nutritious crop and contain considerable amounts of vitamins and minerals. Millets are a good source of energy, dietary fiber, slowly digestible starch, and resistant starch, and thus provide sustained release of glucose and thereby satiety [5,6]. Compared to cereals, millets are a good source of protein- and sulphur-containing amino acids (methionine and cysteine) and have a better fatty acid profile [5,7]. However, millets contain a limited amount of lysine and tryptophan, which varies with the cultivar. Millets are rich in vitamin E and vitamin B and in minerals such as calcium, phosphorus, magnesium, manganese, potassium, and iron [1,8]. The abundant nutrients of millets provide multiple benefits such as reducing the incidence of cancer [9,10], obesity and diabetes [11], cardiovascular diseases [12,13], gastrointestinal problems [14], migraine, and asthma [1,15]. Consumption of millets helps manage hyperglycemia due to their lente carbohydrate and high dietary fiber content, thus making millets a perfect food for the diabetic populace [3,15]. Therefore, millets play an important role in the modern diet as a potential source of essential nutrients, especially in underdeveloped and developing countries [16]. Although millets have a diversified and high food value, their consumption, especially by the Indian populace, has not reached a significant level due to various factors, depicted in Figure 1. Recently, these grains have been slowly fueling the start-up revolution to improve nutri-rich food availability and create employment.

Millets are usually processed before consumption to remove the inedible portions, extend the shelf life, and improve nutritional and sensory properties. Primary processing techniques such as dehulling, soaking, germination, roasting, drying, polishing and milling (size reduction) are followed to make millets fit for consumption. At the same time, modern or secondary processing methods such as fermenting, parboiling, cooking, puffing, popping, malting, baking, flaking, extrusion, etc., are used to develop millet-based value-added processed food products [8]. Although these processing techniques aim to enhance the digestibility and nutrient bioavailability, a significant amount of nutrients are lost during subsequent processing [18]. This review article aims to provide an overview of the effect of processing techniques on the nutritional properties of important Indian millets, viz. pearl millet, proso millet, kodo millet, foxtail millet, and little millet.

## 2. Methodology

Review was conducted based on the methodology reported earlier with slight modification [19]. The current topic was selected based on a literature survey to identify the gap between the available literature resources pertaining to the effect of processing treatment on specific nutrient components of millet with respect to the Indian scenario. The objective of the review was to evaluate the millet processing treatments in order to identify the appropriate processing treatment for maximum retention of nutrients. The review includes peer-reviewed research articles published in the English language after the year 2016. The articles exclusive to dehulling, fermenting, germination, parboiling, cooking, puffing, popping, malting, and extrusion millet processing were included. The literature review was carried out using databases such as PubMed and Google Scholar as search engines. The common search terms used were millets processing, millet nutrition, dehulling, nutri-cereals processing, value addition to millets, fermenting, germination, parboiling, cooking, puffing, popping, malting, extrusion of millets, etc.

## 3. Nutritional Characteristic of Selected Indian Millets

### 3.1. Nutritional Profile of Millets

The nutritional content of food is an important factor in the maintenance of a human body’s metabolism and wellness. The nutritional content is critical for developing and maximizing the human genetic potential. Millet’s nutrition is comparable to major staple cereals (rice, wheat, and maize), since they are an abundant source of carbohydrates, protein, dietary fiber, micronutrients, vitamins and phytochemicals. Millets provide energy ranging from 320–370 kcal per 100 g of consumption (Table 1). Millets have a larger proportion of non-starchy polysaccharides and dietary fiber compared to staple cereals and comprise 65–75% carbohydrates. Millets with high dietary fiber provide multiple health benefits such as improving gastrointestinal health, blood lipid profile, and blood glucose clearance. Millets with minimal gluten and low glycemic index are healthy options for celiac disorder and diabetes [20]. Millets are also rich in health-promoting phytochemicals such as phytosterols, polyphenols, phytocyanins, lignins, and phyto-oestrogens. These phytochemicals act as antioxidants, immunological modulators, and detoxifying agents, preventing age-related degenerative illnesses such as cardiovascular diseases, type-2 diabetes, and cancer [1]. A study [21] reported that millets contain about 50 different phenolic groups and their derivatives with potent antioxidant capacity, such as flavones, flavanols, flavononols, and ferulic acid. A significant amount of phenolic components, which are important antioxidants in millets, are found in bounded form in proso and finger millet and in free form in pearl millet [22]. Another study [23] reported that proso millet comprises various phytochemicals such as syringic acid, chlorogenic acid, ferulic acid, caffeic acid, and p-coumaric. It has also been reported that almost 65% of the phenolics are present in the bound fraction. The presence of these phytochemicals and important antioxidants indicates the potential benefits of millets to human health. A detailed summary of the nutritional profile of selected Indian millets is discussed below and highlighted in Table 1.

- Proso millet has a higher nutritional value when compared with staple cereals as it contains a higher concentration of minerals and dietary fiber (Table 1). Proso millet is a rich source of vitamins and minerals such as iron (Fe), calcium (Ca), potassium (K), phosphorus (P), zinc (Zn), magnesium (Mg), vitamin B-complex, niacin, and folic acid. Proso millet contains essential amino acids in significantly higher quantities, except for lysine, the limiting amino acid. However, proso millet has an almost 51% higher essential amino acid index than wheat [24]. Moreover, the products prepared from proso millet exhibit a lower glycemic response than staple cereal-based products. A review reported that products prepared from proso millet show a significantly lower glycemic index (GI) compared to wheat- and maize-based products [25].
- Pearl millet shows an energy value comparable to the staple cereals. Pearl millet contains a lesser amount of carbohydrates than the staple cereals, and it mainly contains high amylose starch (20–22%), and the insoluble dietary fiber fraction helps in exhibiting a lower glycemic response. Pearl millet protein is gluten-free and contains a higher prolamin fraction, making it suitable for people with gluten sensitivity. The amino acid score in pearl millet is good; however, it is poor source of lysine, threonine, tryptophan, and other sulphur-containing amino acids [23,26]. Pearl millet is high in omega-3 fatty acids and also important nutritional fatty acids such as alpha-linolenic acid, eicosapentaenoic acid, and docosahexaenoic acid. It also contains other micronutrients such as Fe, Zn, copper (Cu), K, Mg, P, manganese (Mn), and B-vitamins [23].
- Kodo millet provides an energy value similar to the other millets and staple cereals. However, with the exception of finger millet, the protein content of kodo millet is lower than that of other selected millets and it provides gluten-free protein (Table 1). Kodo millets contains high amounts of vitamins and minerals, especially B-complex vitamins, B6, niacin and folic acid, Fe, Ca, Mg, K, and Zn. Kodo millet is very easy to digest and thus can be beneficial for infant and geriatric product formulation.
- Foxtail millet has a greater nutritional value compared to major cereals such as wheat and rice due to its copious dietary fiber content, resistant starch, vitamins, minerals, and essential amino acids, except for lysine and methionine, but it is richer than most cereals. Among the selected millets, foxtail millet contains the highest protein (Table 1). Foxtail millet also contains a high amount of stearic and linoleic acids, which helps in maintaining a good lipid profile.
- Finger millet has the highest carbohydrate content among the selected millets. However, carbohydrates consist primarily of slowly digestible starch, dietary fiber, and resistant starch and thus offer a low glycemic index compared to most common cereals such as rice and wheat [27]. Finger millet contains around 7% protein (Table 1), which is less than that of other millets, but it has a good amino acid score and contains more threonine, lysine, and valine than other millets. Subsequently, micronutrients such as Ca, Fe, Mg, K, and Zn, as well as B-vitamins, especially niacin, B6, and folic acid, are abundantly available.
- The nutritional value of little millet is comparable to other cereal and millet crops. It contains around 8.7% protein and balanced amino acids, and it is a rich source of sulphur-containing amino acids (cysteine and methionine) and lysine, which is lacking in most cereals [28]. It is generally considered to induce a lower glycemic response due to the presence of abundant dietary fiber, resistant starch, and slowly digestible starch [29]. It is also a good source of micronutrients such as Fe, P, and niacin. Recently, many value-added products have been prepared using little millet to capitalize on the health benefits of little millet.

**Table 1:** Nutritional profile of millets in comparison with cereals (per 100 g).

Grains	Energy (kcal)	Protein (g)	Carbohydrate (g)	Starch (g)	Fat(g)	Dietary Fiber (g)	Minerals (g)	Ca (mg)	P (mg)
Sorghum	334	10.4	67.6	59	1.9	10.2	1.6	27	222
Pearl millet	363	11.6	61.7	55	5	11.4	2.3	27	296
Finger millet	320	7.3	66.8	62	1.3	11.1	2.7	364	283
Proso millet	341	12.5	70.0	-	1.1	-	1.9	14	206
Foxtail millet	331	12.3	60.0	-	4.3	-	3.3	31	290
Kodo millet	353	8.3	66.1	64	1.4	6.3	2.6	15	188
Little millet	329	8.7	65.5	56	5.3	6.3	1.7	17	220
Barnyard millet	307	11.6	65.5	-	5.8	-	4.7	14	121
Maize	334	11.5	64.7	59	3.6	12.2	1.5	8.9	348
Wheat	321	11.8	64.7	56	1.5	11.2	1.5	39	306
Rice	353	6.8	74.8	71	0.5	4.4	0.6	10	160

Source: Indian Food Composition Tables and nutritive value of Indian foods [30,31].

### 3.2. Antinutrient Profile of Millets

Antinutrients are phytochemical compounds that plants produce naturally for their defense. These antinutritional factors hinder nutrient absorption, leading to reduced nutrient bioavailability and utilization [32]. When consumed uncooked, products containing antinutrients and chemical compounds may be detrimental or even pose health issues in humans, such as micronutrient malnutrition, nutritional deficiency, and bloating. Plant-based foods mainly contain antinutrients such as tannins, phytates, oxalates, trypsin, and chymotrypsin inhibitors [33]. One of the disadvantages of millets is a higher concentration of antinutritional factors compared to wheat and rice. Finger millet contains polyphenols, tannins (0.61%), phytates (0.48%), trypsin inhibitors, and oxalates, which may interfere with the bioavailability of micronutrients and protein digestibility. The goitrogenic compounds in pearl millet are derivatives of phenolic flavonoids, such as C-glycosyl flavones, and their metabolites are responsible for the development of off-odors in the flour during storage [34]. Antinutritional factors due to metal chelation and enzyme inhibition capacity decrease nutrients bioavailability, mainly of minerals and proteins. However, in recent years, antinutritional factors such as polyphenolic compounds have been reported as nutraceuticals for their contribution to antioxidant properties [1]. Most secondary metabolites that function as antinutrients may cause extremely detrimental biological reactions, while others are actively used in nutrition and pharmacologically active drugs. The need of eliminating antinutrients is fulfilled by pretreatment or processing techniques of food grains, such as debranning, soaking, germination, fermentation, and autoclaving. These methods add value to food by enhancing the bioavailability of a few cations such as Ca, Fe, and Zn and also the proteins absorption [8].

## 4. Mechanical Processing for Millets

**Figure 2:**
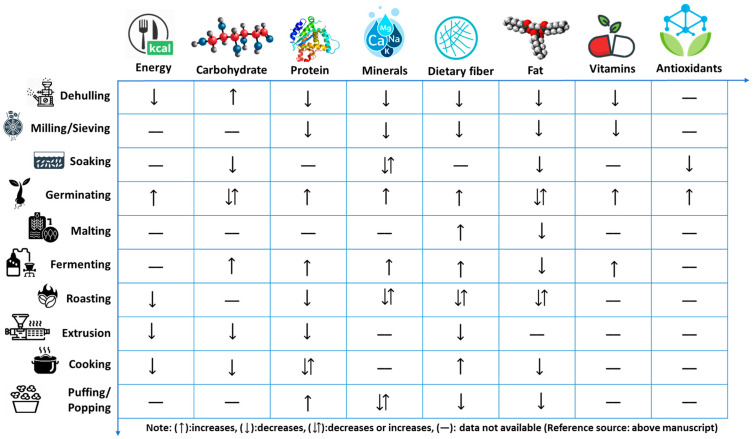
Inference on nutritional properties changes during different processing methods.

Because global food security is at risk, effective utilization of available millet crops to develop an affordable, palatable, and nutrient-rich product is the need of the hour. Millet grains must be processed to remove inedible portions and convert them into cooked and edible form. Therefore, processing is a crucial task, as it increases the bioavailability of nutrients and organoleptic properties and decreases antinutrients [1]. Processing involves multiple techniques such as dehusking/decortication, milling, soaking, germination, fermentation, malting, cooking, and roasting. These operations cause changes in physicochemical attributes that alter the nutrition, function, and physical characteristics of food [15]. Processing may be of two types, namely, primary and secondary processing. Processes such as cleaning, washing (soaking/germination), dehulling, milling (into flour and semolina), and refining to remove the undesired seed coat and antinutritional factors are termed as primary processing, while secondary processing involves converting primary processed raw materials into “ready-to-cook” (RTC) or “ready-to-eat” (RTE) products by flaking, popping, extrusion, and baking [1]. The traditional processing technologies include debranning, milling, roasting, soaking, steaming germination, popping, flaking, ready-to-eat salted grains, and fermented products [35,36]. These processing techniques aim to convert grains into edible forms, with an extended shelf life, improved texture, specific flavor, taste, as well as improved nutritional quality and digestibility [37]. Millet consumption and utilization can be increased by processing them into various by-products, which also reduces the phytate and tannin levels, increases the minerals and amino acids bioavailability, and improves starch and protein digestibility [38]. Processing imparts specific morphological, anatomical, or modulated changes in these bioactive compounds present in whole grains. The processing methods may have positive as well as negative impacts on the nutrient and antinutrient profile. Various research studies on millet processing have shown positive results on the effective usage of millets in a variety of traditional and convenience health foods. Significant levels of phytates, tannins, phenols, and trypsin inhibitors decrease nutrient bioavailability and quality, limiting maximum utilization of nutritional potential in millets [1]. Certain millets contain higher concentrations of unsaturated fatty acids; hence rancidity and off-flavors occur in millet flour during storage due to lipolysis followed by oxidation of “de-esterified fatty acids” [32]. Thus, understanding the influence of processing on nutritional properties is extremely important for effective utilization of millets. It also assists in choosing an appropriate processing technique for millets to maximize nutrient availability, improve palatability, and increase shelf life. The changes in nutritional composition and digestibility with respect to different mechanical processing methods are discussed (Table 2) and summarized (Figure 2).

## 5. Effect of Processing on Nutritional Properties of Millets

### 5.1. Proteins

Millets are a rich source of proteins and are widely consumed by vegans. They are regarded as an excellent plant protein with negligible amounts of saturated fats compared to animal proteins. The presence of antinutrients inhibits protein digestibility; hence, reducing the antinutrients level is important. Simple techniques such as dehulling, milling, soaking, and heating decrease the antinutrient levels and increase the in vitro protein digestibility. The impact of various processing methods on the protein digestibility of foxtail millets has been studied [20]. The alkaline cooking, fermentation, germination (40 h at 25 °C), and popping of foxtail millet resulted in improved protein quality. In another study, pan-frying showed increased protein content in proso millet by 9.5% [18]. The puffing or popping of kodo millet increased the protein concentration from 7.92 to 8.12% [53]. The separation of starch granules from the protein matrix during thermal treatment, as well as the destruction of antinutritional components such as trypsin inhibitors and phytate acid, resulted in enhanced protein digestibility as a result of heat treatment or high pressure.

Protein digestibility in cereals, millets, and legumes has been shown to improve throughout the germination and fermentation processes. The germination of foxtail millet resulted in an increment in the protein concentration due to the synthesis of new amino acids [39]. Similar results for the increase of protein during germination of two cultivars of pearl millet, namely Gadarif (11.4% to 13.2%) and Gazeera (14.4% to 16.3%) were observed [54]. A study [55] showed that following germination, the protein concentration of pearl millet increased from 14% to 26%, whereas another study [43] reported the increased protein in proso millet after sprouting for 96 h. A research study on the impact of fermenting pearl millet flour with pure cultures revealed enhanced protein efficiency ratios, true and apparent protein digestibility, and utilizable protein values [55]. In another study, the combined effect of germination, fermentation (12 h and 24 h, respectively) and dry heating of pearl millets resulted in improved “in vitro protein digestibility” (IVPD), indicating that fermentation enhances protein digestibility [54]. The natural fermentation of pearl millet may significantly enhance the protein content [47]. During fermentation, antinutritional factors such as phytate gets degraded and the insoluble protein get converted to soluble protein due to the synthesis of proteolytic enzymes by microflora [56]. The simple technique of soaking pearl millet for 24 h resulted in increased protein due to the mobilization of stored nitrogen [46]. Similarly the malting of pearl millet (24 h soaking, followed by 18 h germination) significantly enhanced the protein [43]. These reports suggest that the soaking, malting germination, and fermentation processes lead to an increment in the total protein and improved protein digestibility, and thus can be used as an effective processing treatment in the development of protein-rich foods. Because these processes do not necessitate sophisticated equipment, they can be employed at the domestic level as well, assisting in the fight against protein–energy malnutrition, which is primarily a concern in underdeveloped nations.

Decortication removes about 12% to 30% of the outer husk, bran, and germ portion of grains, limiting the significant loss of proteins and amino acids such as histidine, lysine, and arginine. According to a study [49], dehulling of pearl millet up to 17.5% had a significant impact on the nutritional contents, increasing protein and digestibility. However, dehulling beyond this point, a substantial decrease in protein occurred. In another study [57] on the milling of pearl millet, bran-rich milled grains showed the highest percentage of IVPD. Similar improvements in millet’s IVPD were reported by other authors [53]. Since most of the polyphenolic compounds and antinutrients which precipitate proteins and reduce protein digestibility are present in the hull of millets, the decortication process substantially eliminates them and result in improved protein digestibility.

### 5.2. Carbohydrates

Carbohydrates of the millets range around 60–75%, with foxtail millet containing the minimum carbohydrate and little millet containing the maximum carbohydrate (Table 1). Starch is the principal carbohydrate of the millets like other cereals. The amount of available carbohydrates in food grains is affected by various domestic processing and cooking methods such as soaking, sprouting, pressure cooking, autoclaving, and so on [1]. The carbohydrate content of foxtail millet increased significantly, by 1.29% [58]. By contrast, the carbohydrates of pearl millet flour increased non-significantly during the first 24 and 48 h of germination but decreased significantly after 72 h [45]. The increase in carbohydrates during the germination of foxtail millet is associated with the decrease in moisture, ash, crude protein, and fat, because the carbohydrate levels depend on these attributes of the grains [58]. The effect of fermentation and germination on the carbohydrates of pearl millet revealed that germination greatly increases the total soluble sugar concentration, as well as the reducing and non-reducing sugar concentration. When homogenized and autoclaved, the germinated slurry substantially increased the soluble sugars and decreased starch [49,59]. The main reason for reduced starch could be due to the starch hydrolysis during the germination and autoclaving process, resulting in a higher concentration of soluble sugars. In a similar study, fermented pearl millet grains also showed lower levels of starch and higher levels of soluble carbohydrates than native pearl millet grain [60]. Another study revealed a significant rise in the total amount of sugars in proso millet during germination, which could be attributed to starch breakdown [61]. These results indicate that the germination and fermentation processes improve the carbohydrate digestibility by breaking down the complex starch into simple soluble sugars. This shows the importance of germination and fermentation in the development of energy-dense, easily digestible food products such as infant formula. A study [62] reported the effect of decortication and hydrothermal processing on finger millet. They observed that decortication significantly increased the total carbohydrates by around 16%. The reduction in carbohydrates due to decortication is apparent due to the removal of the seed coat. However, no change in total carbohydrates due to hydrothermal treatment was reported, but a slight change in amylose fraction was noted. Furthermore, due to leaching during steeping and the Maillard process during steaming, the sugar concentration reduced from 1.085 to 0.71 g/100 g after hydrothermal processing. These results indicate that carbohydrates behave differently with different processing techniques. An extensive study [32] on the starch digestibility of pearl and proso millet revealed that parboiling significantly reduced the total starch by 5–10% due to starch leaching out during soaking and boiling process. They also observed that parboiled proso and pearl millet had a reduced readily digestible starch fraction (18.2–19.1% to 17.4–18.3%) and thus a lower glycemic index by 1.6–3.9%. These results suggest that parboiling can significantly reduce starch digestibility and therefore can be utilized to formulate products for metabolic diseases such as diabetics and obesity.

### 5.3. Dietary Fiber

The millet bran fraction is a major and abundant source of dietary fiber, which is characterized as complex polysaccharides that are not readily available. Therefore, removal of the bran fraction during decortication/dehulling results in substantial reduction in fiber component. It was reported that dehulling of about 12% to 30% to remove the kernel is suitable for millet grains as it does not result in significant loss of fiber. However, dehulling of grains beyond 30% results in the substantial loss of dietary fiber [37]. Since most of the millets are consumed in their decorticated form, it is very important to control the extent of dehulling so as to maximize the fiber content. A study [20] on the impact of milling on the fiber components of foxtail millet revealed that the insoluble dietary fiber content of lignin, cellulose, and hemicellulose in the milled fraction was lower than that of whole millet flour, while in foxtail millets the fiber content increases significantly with increasing germination time [39]. This is perhaps due to a change in the structure of the seeds’ cell wall polysaccharides, which may affect the tissue histology and disrupt protein carbohydrate interactions. In addition, the results of cell wall biosynthesis leads to increased production of dietary fiber. A study of solid-state fermentation (SSF) on pearl millet with *Rhizopus oligosporus* and *Yarrowia lipolytica* [63] increased the soluble dietary fiber by 176%. Another study revealed that, fermenting the dietary fiber from foxtail millet bran with *Bacillus natto* enhanced the soluble dietary fiber (DF) content by 10.9% and increased the ratio of soluble DF to insoluble DF by 16.8% [64]. Following fermentation, cellulose and hemicellulose breakdown resulted in more porous structure polysaccharides, which explains the changes in DF. Similarly, malting pearl millet for 24 h boosted the fiber level from 0.77% to 0.87% [44]. A study [65] on maize and finger millet-based extruded product showed that the non-starchy polysaccharides reduced from 2.5 g/100 g for raw blend to 1.5 g/100 g for unfermented-extruded blend. The values were further reduced to 0.9 for fermented blends and 1.4 g/100 g for blends treated with lactic or citric acid (different molarities) prior to extrusion. It was also observed that high extrusion temperatures and severe mechanical shear disrupt glycosidic networks and weak bonds between polysaccharide chains of dietary fiber polysaccharides, resulting in a reduction in total NSP. Similarly, the thermal processing of biscuits prepared from pearl millet flour resulted in a change in crude fiber content from 1.26% to 1.75% [63]. Roasting of pearl millet grains at different times and temperatures reduced crude fiber content. Other thermal processes such as puffing and popping on millets resulted a decline in crude fiber by 1.71% and from 18.9 to 15.8 g/100 g, respectively [66]. This could be mainly attributed to the fact that the outer grain layer has the majority of the fiber that is exposed to thermal degradation. To summarize, the reports suggest that dehulling and milling (debranning) operations reduce dietary fiber, while high temperature extrusion processes lead to thermal degradation of dietary fiber. Dietary fiber, particularly that accumulated in the outer bran layer, plays a vital role in reducing type 2 diabetes and constipation. For a healthy millet diet, it is important to discourage millers from polishing millets and to advise consumers to prefer whole millets (unpolished) and their by-products.

### 5.4. Minerals

Millets are an abundant source of minerals such as K, Mg, Fe, Ca, and Zn, along with vitamins that are mainly accumulated in the aleurone, germ, and pericarp [1]. Soaking millet grains prior to cooking helps to reduce antinutrients while also improving mineral bioavailability. Millet grains soaked in water were shown to have reduced Zn and Fe content, which might be attributed to minerals leaching into the soaking water [67]. Soaking millet grains boosts the “in vitro solubility” of minerals such as Fe and Zn by 2–23%. Soaking the millet grains in hot water (45 to 65 °C) with a pH of 5–6 resulted in a significant increase in bioavailability and a decrease in phytic acid [68]. The mineral content in pearl millet flour was affected by germination and fermentation [49]. Germination of foxtail millet improved and modified the nutrient profile by increasing the mineral compounds availability [20,49]. Germination increased the availability of minerals by the catabolism process of antinutrients such as saponins and polyphenols, which inhibit the mineral bioavailability [39]. A similar increase in the mineral concentration in germinated foxtail millet was reported [69]. Germination also activate phytase-specific phosphatases enzyme called phytases, which hydrolyze phytate into inositol and orthophosphate and release minerals. Therefore, increased levels of minerals such as Mg (101.16 to 107.16 mg/kg), sodium (Na) (63.34 to 69.45 mg/kg), Ca (17.43 to 25.62 mg/kg), and Fe (16.01 to 54.23 mg/kg) were reported for foxtail millet [39]. The mineral content of kodo millet increased from 232.82 to 251.73 mg/100 g after 36 h of germination at 38.75 °C [41]. According to [70], fermentation improved the availability of Ca by 20%, Fe by 27%, and P and Zn by 26%. Bleaching pearl millet for 90 s increased Fe availability from 2.19 to 3.29 mg/100 g in vitro [49].

The decorticated millet grains decreased the total mineral content: Ca by 40%, Fe by 50%, and Zn by 12%; however, it increased the bio-accessibility of the minerals Ca (15 g/100 g), Fe (26 g/100 g), and Zn (24 g/100 g) [53]. The decortication process reduces the antinutrients, which inhibit mineral bioavailability by creating complexes. The antinutrient level reduction leads to an improvement in the bioavailability of minerals [53]. Another study discovered that the whole grain flour of foxtail millet after milling was mineral-rich, while the polished grain flour showed reduced mineral content but with a higher protein content [20]. Semi-polished pearl millet has been shown to significantly reduce ash content (1.5% to 1.3%), which represents the noncombustible portion of minerals. The decrease in the ash content was associated with removal of bran. Minerals such as Ca and P, along with antinutrients, are accumulated in the bran fraction of pearl millet [70]. However, semi-refining reduces the phytate content, which results in improved in vitro bio-accessibility of Fe and Ca. Milling and sieving of finger millet caused a reduction in some minerals such as Fe (6.52 to 3.29 mg), Zn (2.50 to 1.98 mg), and Ca (404.3 to 294.8 mg) [71].

The total Fe content of roasted pearl millet grains increased by 274 percent, which was due to leaching from the roasting iron-pan into millet samples during the high-temperature roasting process [72]. Similar studies on finger millet roasting increased the minerals such as Ca (337.31 to 341.24 mg/100 g) and Fe (3.45 to 3.91 mg/100 g) [73]. Foxtail millets processed through solid-state fermentation (SSF) were rich in important minerals and amino acids [63]. The mineral content was enhanced when fermented foxtail millet flour was incorporated with a single strain of *L. acidophilus* [20]. Studies also indicate that pure culture fermented products increase the bioavailability of minerals [53].

The dark gray color of pearl millet grains restricts their usage in food preparation. This drawback can be overcome by treating millet grains with organic acids (fumaric, acetic, and tartaric acid) or natural acidic materials (tamarind). Various researchers have studied the effect of acid treatment. A study on acid treatment, which includes soaking the grains in 0.2 N HCl solution for 24 h, subsequent washing, blanching (98 °C for 30 s), and sun-drying (2 days), significantly improved the P, Ca, and Fe extractability [74]. This increase in HCl extractability was accompanied by an increase in mineral bioavailability. When compared to native grains, pearl millet treated with acid for 18 h significantly improved the in vitro Fe bio-accessibility. The Fe concentration decreased because of the leaching of minerals naturally accumulated in the pericarp portion during processing [49,53]. The millet-based composite flour incorporated with skimmed-milk powder and vegetables showed a substantial increase in Zn (2.1–4.2 mg/100 g), Ca (143.6–667.8 mg/100 g) and Cu (0.5–0.9 mg/100 g), but no significant changes in Fe (3.4–3.6 mg/100 g) and Mg (4.3–4.4 mg/100 g) [75]. The report suggests that the majority of minerals are accumulated in the germ and bran layer which will be lost during dehulling and sieving operations. However, the process of germination and fermentation was found to increase the mineral content to some extent which could be exploited to develop value-added products.

### 5.5. Vitamins

Millets when polished/debranned contain a lower nutritional value since the bran and germ components of refined millet flour are eliminated, resulting in a loss of vitamins. Millets are considered superior to wheat, sorghum, and maize in terms of vitamin content and other nutrients that include fats, proteins, and minerals (Table 1). Vitamins along with minerals are naturally accumulated in the aleurone, germ, and pericarp.

Millet grains are high in vitamins such as riboflavin, thiamine, niacin, and folic acid [76]. It has been noted that the germination and fermentation processes in pearl millet affect the vitamin content of the grains. Improved vitamin levels (thiamin) after the fermentation process were reported [49]. Little millet decortication resulted in a 67% reduction in vitamin E [77]. The milling affects the bran portion of the millet grains, which reduces vitamins that are mainly accumulated in the outer bran layer of grains. Milling pearl millet grains resulted in a considerable decrease in vitamin B and a modest reduction in vitamin E, but milling and sieving of finger millet flour tends to decrease vitamins such as thiamine (0.552 to 0.342 mg/100 g) and riboflavin (0.243 to 0.196 mg/100 g) [71]. The germination of finger millet showed increased vitamin C content, from 0.04 to 0.06 mg/100 g [66]. Similarly, increased levels of vitamins (thiamine, niacin) after germination and probiotic fermentation were reported [49,55]. The elevation of some vitamins levels, especially thiamine, niacin, and riboflavin, was observed during finger millet fermentation [78]. Biscuits prepared by replacing refined wheat flour with 45% of foxtail millet flour resulted in an increased value of vitamin content such as niacin (1.41%) and thiamin (0.1836%), except riboflavin (0.09%) [79]. The nutritional and storage characteristics of nutritious millet food of the West African region were studied. It was found that vitamin B2 concentration was likely reduced by 31.4%, 34.3%, and 45.7% after the processing of grain to a meal, flour, and fura, respectively [55]. The studies on milling or dehulling suggest that the vitamins are lost during these processing operations as the majority of vitamins are accumulated in the outer layer of millets. The availability of important vitamins can be improved by germinating the millets and developing by-products from germinated millets.

### 5.6. Fats

Fats are necessary for calorie supply, brain development, and the absorption and transport of vitamins A, D, E, and K in the body. The germination time has an impact on fat content. For instance, the raw and optimized flour of germinated foxtail millet had 4.4% and 3.6% fat, respectively which was substantially lower than the non-germinated sample. This is due to the fact that the fat is used as an energy source throughout the germination process, which leads to the reduction after germination [39]. A study to investigate the effect of high-pressure soaking on the nutritional characteristics of foxtail millet revealed that the fat content is reduced by 27.98% [40]. This was attributable to the enzymatic activity that creates free and soluble nutrients throughout the germinated phase in foxtail millets. Similarly, another study reported that malting of pearl millet for 24 h resulted in a reduction in fat by 6.34 to 5.55% [44]. During germination the increased enzyme and fat consumption as an energy source might explain the reduction in fat content. According to a study on the influence of different cooking techniques on the characteristic changes of foxtail millet [18], the fat content was highest in the roasted sample (3.2 g), followed by the raw (2.9 g), pressure cooked (2.8 g), germinated (2.6 g), and boiled sample (1.9 g). The effect of pearl millet fermentation on crude fat, reduced its value from 2.25 to 1.70% [63]. Another study on fermentation of pearl millet reported an increase in crude fat content from 1.83 to 3.71% [37,49]. Germination of foxtail millet was found to reduce the fat content, which is related to lipid hydrolysis and fatty acid oxidation that occurs during germination [55]. The foxtail millet grains were germinated at 30 °C and little millet at 35 °C for 24 h after overnight steeping, then tray dried at 60 °C for 6 h and milled for further analysis. The fat content reduced by 17.84% in foxtail millet and increased in little millet by 25.95% [58]. This was due to the changes in energy values since the fat content includes approximately double the energy values of protein and carbohydrate.

Thermal processing of biscuits made from pearl millet flour resulted in a percentage change in crude fat content from 2.25 to 18.77% [63]. Another study focused on thermal processing such as pan cooking and microwave heating on proso millet results showed a decreased level of fat content from 3.24 to 2.3 g/100 g (pan cooking) and from 3.24 to 3.05 g/100 g (microwave cooking), while for little millet, fat content decreased from 1.91 to 1.56 g/100 g (pan cooking) and from 1.91 to 1.79 g/100 g (microwave cooking) [52]. Similarly, roasting decreased the crude fat content by 0.71%, puffing and popping decreased fat content by 0.06% and 1.3–0.63 g/100 g, respectively [66]. The study on the popping of foxtail millet reported having lower value of crude fat content than raw millet [55]. Bleaching of pearl millet for 90 s resulted in a greater drop in free fatty acids level from 44.56 to 20.59 mg/100 g [49].

The use of roller mills for the production of low-fat pearl millet grits was investigated, and it was observed that decortication, tempering, and milling using finer corrugated rollers offered an average output of 61% grits (from whole grains) and 1.2% fat content [49]. By contrast, another study stated that decortication of pearl millet had no significant changes in fat content. It was also observed that when moisture content and milling time increase, the fat, ash, and fiber content reduces [55]. Development of composite millet flour had a higher rate of oil and water absorption capacity than that of millet flour [75]. The oil absorption capacity (OAC) and water absorption capacity (WAC) of the composite flour of different millets increased from 59.2% to 77.9% and from 117% to 225%, respectively. The OAC refers to flour protein’s capacity to physically bind fat through capillary attraction, which is essential since fats function as flavor retainers and improve the mouthfeel of foods. The studies provide sufficient evidence on degradation or denaturation of fat at high temperature processing (cooking and popping) as well as reduction in fat content during milling, malting and fermentation processes. The simple processing techniques such as soaking, germination and malting could be the ideal option for manufacturers to develop low-fat food products from millets. The high temperature processing would damage the fat quality and might reduce the taste and flavor of the processed foods. 

## 6. Conclusions

Millets have an energy value similar to staple cereals. Additionally, they provide more significant health benefits due to their high fiber, minerals, vitamins, macro- and micronutrients, and phytochemicals and can help combat chronic disorders. Making millets part of a regular diet can provide an affordable, complete, and healthy meal. It was observed that during germination and fermentation of millets, the dietary fiber, mineral, and vitamin content of most millets improved. Simple processing techniques such as soaking, germination/malting, and fermentation can help tackle the problem of protein–energy malnutrition by improving protein digestibility and the bioavailability of the minerals. However, it was observed that decortication, dehulling, milling, extrusion resulted in a reduction of total proteins, total dietary fiber, and micronutrients. Thus, care should be taken during the decortication of millets, as excessive dehulling can result in lower fiber content and loss of micronutrients due to the loss of nutrient-rich bran and germ portion. 

Looking into the variability of the impact of processing on the nutritional characteristics of millets, there is still a need to focus on optimizing the processing techniques for minor millets to make them more acceptable without compromising the health benefits. Moreover, to combat food insecurity and malnutrition, awareness needs to be created at both commercial and household levels regarding the impact of processing methods on the nutritional properties of millets and the health benefits of millets.

## Figures and Tables

**Table 2:** Changes in millets nutritional properties with respect to processing methods.

Processing Methods	Millets	Experimental Condition	Inference/Study Outcome	References
Germination	Foxtail	Germinated for 46.5 h (optimized)	Increased protein content (13.75 g/100 g) as compared to raw seeds (10.60 g/100 g). Increased dietary fiber by 5.2%. Elevated the levels of minerals such as Fe, Mg, Ca, and Na. Free, bound, and total phenolics and flavonoids content is increased. Decreased fat content from 3.86 to 2.78 g/100 g.	[39,40]
		Germination at room temp with tap water	Increased protein (by 29.72%) total dietary fiber (58.02%) and total phenolic content (77.42%). Increased level of DPPH radical scavenging was observed.	[20,35]
	Kodo	38.75 °C for 36 h	Elevation in mineral content. Protein and dietary fiber content increased. Total carbohydrates reduced.	[41]
	Pearl Var; Kalukombu (K) and Maharashtra Rabi Bajra (MRB)	Sprouting at room temperature for 72 h	Protein content reduced in MRB variety, while K variety had no significant effect. Fat and ash content reduced. Iron and calcium content significantly increased after germination.	[42]
	Proso	Sprouting for 96 h	Protein and minerals become more biologically accessible.	[43]
Malting	Pearl Var; Ex-Borno	Steeping at 25 °C for 24 h, Germinated at different time intervals, kilned (hot-air oven) at 55 °C for 18 h	Protein content increased from 7.52% (control) to 9.19% (96 h) malted millet flour. Crude fiber increased with an increasing malting period (i.e., 0.77% for control to 1.38% for 96 h malted sample). Decrease in carbohydrate content due to starch hydrolyzed into simple sugars by enzymes such as α- and β-amylase. Fat level was found to be lowest for 96 h of malted samples, which affects energy values of millet flour, but ensures increased shelf life. Kilning and steeping process decreases the level of amino acids (tyrosine, isoleucine, methionine, glycine, cysteine and glutamic acid).	[44]
	Pearl	Alkaline steeping of malted flour (2% Ca (OH_2_) and (2% ash solution))	Both the steeping methods increase the protein level of flour samples. Lime steeped millet flour had increased fiber content as compared to ash steeped and control flours. Lime steeping lowered the levels of crude lipid in millet flour. Ca, Mg, and K levels increased while phosphorus and zinc levels decreased as steeping duration progressed.	[45]
Soaking	Pearl	Soaking for 24 h	Protein content increased due to the mobilization of stored nitrogen of grains. Fat content and crude fiber increases with sprouting The utilization of energy sources results in reduced carbohydrates.Sprouting reduced minerals (Co, Cr, Mn, Cu, Zn, Fe, Na, and K) due to leaching, but Ca content increased due to degradation of phytic acid.	[46]
	Foxtail Var; white	High-pressure soaking (600 MPa, 60 °C and 120 min)	Protein level decreased from 13.65% (native) to 13.11% (treated sample) due to the formation of protein–starch complex.	[40]
Fermentation	Pearl Var; Sosart 1	Pure cultures of Lactobacillus plantarum	Increase in protein content after 96 h fermentation from 8.7% in unfermented sample to 20.54% in starter culture fermented sample and 20.21% in naturally fermented sample. Lipid content decreased from 10.34 to 0.34 (starter culture sample) and 0.74 (naturally fermented). Carbohydrates decreased with a parallel increase in soluble sugar.	[47]
	Foxtail	Fermentation followed by heat moisture treatment	Increased crude protein content. Decreased the total carbohydrate level. Enhanced the nutritional quality of starch.	[20]
		Fermentation using *L. paracasei* Fn032 strain	Crude protein content increased by 20.51% in the fermented sample. Total carbohydrate decreased to 74.02%.	[48]
Cooking/ Boiling/ Roasting	Pearl	Roasting (150 °C for 5 min)	Increased the percentage bio-accessibility of total polyphenols from 73.2% in native grains to 78.1% in roasted samples. Bio-accessible flavonoid content increased.	[49,50]
		Pressure cooking (15 psi in triple distilled water for 15 to 20 min)	Total polyphenol content decreased by 29%.	
		Blanching 98 °C for 10–20 s	Lowered the percentage of free fatty acids, acid value and fat acidity.	
		Microwave heating	Reduced bio-accessibility of phenolic content.	
	Foxtail	Soaking followed by cooking	Maximum decrease in protein, Fe, and Zn. Increased the bioavailability of soluble Zn and ionizable Fe.	[20]
	Kodo	Boiling at 95–100 °C for 25 min	Increased porosity and water absorption capacity. Reduced starch yield.	[38]
		Pressure cooking at 9.8 × 10^4^ Pa for 20 min	High level of resistant starch observed. Enhanced oil absorption capacity.	
		Puffing 230 °C for 3 min	Increased carbohydrate content from 68.35% to 74.38%. Increased protein content from 7.92% to 8.12%. Decreased crude fiber and fat content. Calcium level reduced from 27 to 18 mg/100 g.	[51]
	Proso	Pan and microwave cooking	Increased level of DPPH and FRAP radical scavenging activity. Increased carbohydrate content but decreased fat content. Protein content increased in pan cooking but decreased in microwave cooking.	[35,52]
	Little	Pan and microwave cooking	Carbohydrate content increased, while fat content decreased. Protein content increased in microwave cooking but decreased in pan cooking.	[52]

## Data Availability

Not applicable.

## References

[1] Rao D.B., Malleshi N.G., Annor G.A., Patil J.V. (2017). Nutritional and health benefits of millets. Millets Value Chain for Nutritional Security: A Replicable Success Model from India.

[2] Ashoka P., Gangaiah B., Sunitha N. (2020). Millets-foods of twenty first century. Int. J. Curr. Microbiol. Appl. Sci..

[3] Nainwal K. (2018). Conservation of minor millets for sustaining agricultural biodiversity and nutritional security. J. Pharmacogn. Phytochem..

[4] Dutta M., Selvamani Y., Singh P., Prashad L. (2019). The double burden of malnutrition among adults in India: Evidence from the national family health survey-4 (2015-16). Epidemiol. Health.

[5] Nithiyanantham S., Kalaiselvi P., Mahomoodally M.F., Zengin G., Abirami A., Srinivasan G. (2019). Nutritional and functional roles of millets—A review. J. Food Biochem..

[6] Annor G.A., Tyl C., Marcone M., Ragaee S., Marti A. (2017). Why do millets have slower starch and protein digestibility than other cereals?. Trends Food Sci. Technol..

[7] Anitha S., Govindaraj M., Kane-Potaka J. (2020). Balanced amino acid and higher micronutrients in millets complements legumes for improved human dietary nutrition. Cereal Chem..

[8] Birania S., Rohilla P., Kumar R., Kumar N. (2020). Post harvest processing of millets: A review on value added products. Int. J. Chem. Stud..

[9] Ramadoss D.P., Sivalingam N. (2020). Vanillin extracted from Proso and Barnyard millets induce apoptotic cell death in HT-29 human colon cancer cell line. Nutr. Cancer.

[10] Yang R., Shan S., Zhang C., Shi J., Li H., Li Z. (2020). Inhibitory Effects of Bound Polyphenol from Foxtail Millet Bran on Colitis-Associated Carcinogenesis by the Restoration of Gut Microbiota in a Mice Model. ACS Appl. Mater. Interfaces.

[11] Vedamanickam R., Anandan P., Bupesh G., Vasanth S. (2020). Study of millet and non-millet diet on diabetics and associated metabolic syndrome. Biomedicine.

[12] Hegde P.S., Rajasekaran N.S., Chandra T.S. (2005). Effects of the antioxidant properties of millet species on oxidative stress and glycemic status in alloxan-induced rats. Nutr. Res..

[13] Ren X., Yin R., Hou D., Xue Y., Zhang M., Diao X., Zhang Y., Wu J., Hu J., Hu X. (2018). The glucose-lowering effect of foxtail millet in subjects with impaired glucose tolerance: A self-controlled clinical trial. Nutrients.

[14] Chen Y., Xu J., Ren Q. (2018). The Effect of Millet Porridge on the Gastrointestinal Function in Mice. J. Food Nutr. Res..

[15] Gowda N.N., Taj F., Subramanya S., Ranganna B. (2020). Development a table top centrifugal dehuller for small millets. AMA Agric. Mech. Asia Africa Latin Am..

[16] Anbukkani P., Balaji S.J., Nithyashree M.L. (2017). Production and consumption of minor millets in India-a structural break analysis. Ann. Agric. Res. New Ser..

[17] DES-GOI (2020). Agricultural Statistics at a Glance 2020. (Various Issues). Directorate of Economics and Statistics.

[18] Nazni S., Devi S. (2016). Effect of processing on the characteristics changes in barnyard and foxtail millet. J. Food Process. Technol..

[19] Guiné R.P.F., Barroca M.J., Coldea T.E., Bartkiene E., Anjos O. (2021). Apple fermented products: An overview of technology, properties and health effects. Processes.

[20] Sharma N., Niranjan K. (2018). Foxtail millet: Properties, processing, health benefits, and uses. Food Rev. Int..

[21] Azad M.O.K., Jeong D.I., Adnan M., Salitxay T., Heo J.W., Naznin M.T., Lim J.D., Cho D.H., Park B.J., Park C.H. (2019). Effect of different processing methods on the accumulation of the phenolic compounds and antioxidant profile of broomcorn millet (*Panicum Miliaceum* L.) Flour. Foods.

[22] Chandrasekara A., Shahidi F. (2010). Content of insoluble bound phenolics in millets and their contribution to antioxidant capacity. J. Agric. Food Chem..

[23] Zhang L., Liu R., Niu W. (2014). Phytochemical and antiproliferative activity of proso millet. PLoS ONE.

[24] Kalinova J., Moudry J. (2006). Content and quality of protein in proso millet (*Panicum miliaceum* L.) varieties. Plant Foods. Hum. Nutr..

[25] Das S., Khound R., Santra M., Santra D.K. (2019). Beyond bird feed: Proso millet for human health and environment. Agriculture.

[26] Nambiar V.S., Dhaduk J.J., Sareen N., Shahu T., Desai R. (2011). Potential functional implications of pearl millet (*Pennisetum glaucum*) in health and disease. J. Appl. Pharm. Sci..

[27] Gull A., Jan R., Nayik G.A., Prasad K., Kumar P. (2014). Significance of finger millet in nutrition, health and value added products: A Review. J. Environ. Sci. Comput. Sci. Eng. Technol. Sect. C Eng. Technol..

[28] Neeharika B., Suneetha W.J., Kumari B.A., Tejashree M. (2020). Organoleptic properties of ready to reconstitute little millet smoothie with fruit juices. Int. J. Environ. Clim. Chang..

[29] Patil K.B., Chimmad B.V., Itagi S. (2015). Glycemic index and quality evaluation of little millet (*Panicum miliare*) flakes with enhanced shelf life. J. Food. Sci. Technol..

[30] NIN (2017). Indian Food Compostion Tables.

[31] IIMR (2017). Nutritional and Health Benefits of Millets.

[32] Bora P., Ragaee S., Marcone M. (2019). Characterisation of several types of millets as functional food ingredients. Int. J. Food Sci. Nutr..

[33] Popova A., Mihaylova D. (2019). Antinutrients in plant-based foods: A Review. Open Biotechnol. J..

[34] Taylor J.R.N., Emmambux M.N. (2008). Gluten-free foods and beverages from millets. Gluten-Free Cereal Products and Beverages.

[35] Pradeep P.M., Sreerama Y.N. (2015). Impact of processing on the phenolic profiles of small millets: Evaluation of their antioxidant and enzyme inhibitory properties associated with hyperglycemia. Food Chem..

[36] Rathore T., Singh R., Kamble D.B., Upadhyay A., Thangalakshmi S. (2019). Review on finger millet: Processing and value addition. Pharma Innov. J..

[37] Yousaf L., Hou D., Liaqat H., Shen Q. (2021). Millet: A review of its nutritional and functional changes during processing. Food Res. Int..

[38] Deshpande S.S., Mohapatra D., Tripathi M.K., Sadvatha R.H. (2015). Kodo millet-nutritional value and utilization in Indian foods. J. Grain Process. Storage.

[39] Sharma S., Saxena D.C., Riar C.S. (2015). Antioxidant activity, total phenolics, flavonoids and antinutritional characteristics of germinated foxtail millet (*Setaria italica*). Cogent Food Agric..

[40] Sharma N., Goyal S.K., Alam T., Fatma S., Chaoruangrit A., Niranjan K. (2018). Effect of high pressure soaking on water absorption, gelatinization, and biochemical properties of germinated and non-germinated foxtail millet grains. J. Cereal Sci..

[41] Sharma S., Saxena D.C., Riar C.S. (2017). Using combined optimization, GC–MS and analytical technique to analyze the germination effect on phenolics, dietary fibers, minerals and gaba contents of kodo millet (*Paspalum scrobiculatum*). Food Chem..

[42] Suma P.F., Urooj A. (2014). Nutrients, Antinutrients & bioaccessible mineral content (in vitro) of pearl millet as influenced by milling. J. Food. Sci. Technol..

[43] Morah F.N.I., Etukudo U.P. (2017). Effect of sprouting on nutritional value of panicium miliaceum (Proso Millet). Edorium. J. Nutr. Diet.

[44] Obadina A.O., Arogbokun C.A., Soares A.O., de Carvalho C.W.P., Barboza H.T., Adekoya I.O. (2017). Changes in nutritional and physico-chemical properties of pearl millet (*Pennisetum glaucum*) ex-borno variety flour as a result of malting. J. Food. Sci. Technol..

[45] Bello F.A. (2017). Effect of alkaline steeping on the nutritional, antinutritional and functional properties of malted millet (*Pennisetum glaucum*) flour. Int. J. Innov. Food Nutr. Sustain. Agric..

[46] Iyabo O.O., Ibiyinka O., Abimbola Deola O. (2018). Comparative study of nutritional, functional and antinutritional properties of white sorghum bicolor (Sorghum) and pennisetum glaucum (Pearl Millet). Int. J. Eng. Technol. Man. Res..

[47] Chinenye O.E., Ayodeji O.A., Baba A.J. (2017). Effect of fermentation (Natural and Starter) on the physicochemical, anti-nutritional and proximate composition of pearl millet used for flour production. Am. J. Biosci. Bioeng..

[48] Amadou I., Gounga M.E., Shi Y.H., Le G.W. (2014). Fermentation and heat-moisture treatment induced changes on the physicochemical properties of foxtail millet (Setaria Italica) flour. Food Bioprod. Process.

[49] Rani S., Singh R., Sehrawat R., Kaur B.P., Upadhyay A. (2018). Pearl millet processing: A Review. Nutr. Food. Sci..

[50] Hithamani G., Srinivasan K. (2014). Effect of domestic processing on the polyphenol content and bioaccessibility in finger millet (Eleusine Coracana) and pearl millet (*Pennisetum glaucum*). Food Chem..

[51] Patel A., Vishwa Vidyalaya K., Pradesh M., Parihar P., Ketki Dhumketi I., Anamika Patel C., Dhumketi K. (2018). Nutritional evaluation of kodo millet and puffed kodo. Int. J. Chem. Stud..

[52] Kumar S.R., Sadiq M.B., Anal A.K. (2020). Comparative study of physicochemical and functional properties of pan and microwave cooked underutilized millets (Proso and Little). LWT.

[53] Jaybhaye R.V., Pardeshi I.L., Vengaiah P.C., Srivastav P.P. (2014). Processing and technology for millet based food products: A review nutrient composition of millets. J. Ready Eat Food.

[54] Hassan A.B., Mohamed Ahmed I.A., Osman N.M., Eltayeb M.M., Osman G.A., Babiker E.E. (2006). Effect of processing treatments followed by fermentation on protein content and digestibility of pearl millet (*Pennisetum typhoideum*) cultivars. Pak. J. Nutr..

[55] Saleh A.S.M., Zhang Q., Chen J., Shen Q. (2013). Millet Grains: Nutritional quality, processing, and potential health benefits. Compr. Rev. Food Sci. Food Saf..

[56] Rani M., Amane D., Ananthanarayan L. (2019). Impact of partial replacement of rice with other selected cereals on idli batter fermentation and idli characteristics. J. Food Sci. Technol..

[57] Pushparaj F.S., Urooj A. (2011). Influence of processing on dietary fiber, tannin and in vitro protein digestibility of pearl millet. Food Nutr. Sci..

[58] Kulla S., Hymavathi T.V., Kumari B.A., Reddy R.G., Rani C.V.D. (2021). Impact of germination on the nutritional, antioxidant and antinutrient characteristics of selected minor millet flours. Ann. Phytomed. Int. J..

[59] Pawase P.A., Shingote A., Chavan U.D. (2019). Studies on evaluation and determination of physical and functional properties of millets. (Ragi and Pearl Millet). Asian J. Dairy Food. Res..

[60] Khetarpaul N., Chauhan B.M. (1990). Effect of Germination and fermentation on in vitro starch and protein digestibility of pearl millet. J. Food. Sci..

[61] Balasubramanian S., Sharma R., Kaur J., Bhardwaj N. (2014). Characterization of modified pearl millet (*Pennisetum typhoides*) starch. J. Food. Sci. Technol..

[62] Dharmaraj U., Malleshi N.G. (2011). Changes in carbohydrates, proteins and lipids of finger millet after hydrothermal processing. LWT—Food Sci. Technol..

[63] Kaur P., Purewal S.S., Sandhu K.S., Kaur M., Salar R.K. (2019). Millets: A cereal grain with potent antioxidants and health benefits. J. Food. Meas. Charact..

[64] Chu J., Zhao H., Lu Z., Lu F., Bie X., Zhang C. (2019). Improved physicochemical and functional properties of dietary fiber from millet bran fermented by Bacillus Natto. Food Chem..

[65] Onyango C., Noetzold H., Bley T., Henle T. (2004). Proximate composition and digestibility of fermented and extruded uji from maize-finger millet blend. LWT—Food. Sci. Technol..

[66] Chauhan E.S. (2018). Effects of processing (germination and popping) on the nutritional and anti-nutritional properties of finger millet (*Eleusine coracana*). Curr. Res. Nutr. Food. Sci..

[67] Bindra D., Manju D. (2019). Formulation and evaluation of foods from composite flour blends of sorghum, pearl millet and whole wheat for suitability in diabetic diet. Int. J. Home Sci..

[68] Ertop M., Bektaş M. (2018). Enhancement of bioavailable micronutrients and reduction of antinutrients in foods with some processes. Food Health.

[69] Coulibaly A., Chen J. (2011). Evolution of energetic compounds, antioxidant capacity, some vitamins and minerals, phytase and amylase activity during the germination of foxtail millet. Am. J. Food Technol..

[70] Pushparaj F.S., Urooj A. (2017). Impact of household processing methods on the nutritional characteristics of pearl millet (Pennisetum typhoideum): A Review. MOJ Food Process. Technol..

[71] Oghbaei M., Prakash J. (2016). Effect of primary processing of cereals and legumes on its nutritional quality: A Comprehensive Review. Cogent Food. Agric..

[72] Obadina A., Ishola I., Adekoya O., Soares A., de Carvalho C.W., Barboza H. (2016). Nutritional and physico-chemical properties of flour from native and roasted whole grain pearl millet (*Pennisetum glaucum* [L.] R. Br.). J. Cereal Sci..

[73] Singh N., David J., Thompkinson D.K., Seelam B., Rajput H., Morya S. (2018). Effect of roasting on functional and phytochemical constituents of finger millet (*Eleusine coracana* L.). The Pharma Innov. J..

[74] Arora P., Sehgal S., Kawatra A. (2003). Content and HCl-extractability of minerals as affected by acid treatment of pearl millet. Food Chem..

[75] Tumwine G., Atukwase A., Tumuhimbise G.A., Tucungwirwe F., Linnemann A. (2019). Production of nutrient-enhanced millet-based composite flour using skimmed milk powder and vegetables. Food Sci. Nutr..

[76] Shahidi F., Chandrasekara A. (2015). Processing of millet grains and effects on non-nutrient antioxidant compounds. Processing and Impact on Active Components in Food.

[77] Kamatar M.Y., Kundgol N.G., Math K.K. (2013). Impact of decortication on chemical composition, antioxidant content and antioxidant activity of little millet landraces. Int. J. Eng. Res. Technol..

[78] Konapur A., Gavaravarapur S.R.M., Gupta S., Nair K.M. (2014). Millets in meeting nutrition security: Issues and way forward for India. Indian J. Nutr. Diet..

[79] Anju T., Sarita S. (2010). Suitability of foxtail millet (Setaria Italica) and barnyard millet (Echinochloa Frumentacea) for development of low glycemic index biscuits. Malays. J. Nutri..

